# Developmentally Changing Attractor Dynamics of Manual Actions with Objects in Late Infancy

**DOI:** 10.1155/2018/4714612

**Published:** 2018-11-01

**Authors:** Jeremy I. Borjon, Drew H. Abney, Linda B. Smith, Chen Yu

**Affiliations:** Department of Psychological and Brain Sciences, Indiana University, Bloomington, IN, USA

## Abstract

Human infants interact with the environment through a growing and changing body and their manual actions provide new opportunities for exploration and learning. In the current study, a dynamical systems approach was used to quantify and characterize the early motor development of limb effectors during bouts of manual activity. Many contemporary theories of motor development emphasize sources of order in movement over developmental time. However, little is known about the dynamics of manual actions during the first two years of life, a period of development with dramatic anatomical changes resulting in new opportunities for action. Here, we introduce a novel analytical protocol for estimating properties of attractor regions using motion capture. We apply this new analysis to a longitudinal corpus of manual actions during sessions of toy play across the first two years of life. Our results suggest that the size of attractor regions for manual actions increases across development and that infants spend more time inside the attractor region of their movements during bouts of manual actions with objects. The sources of order in manual actions are discussed in terms of changing attractor dynamics across development.

## 1. Introduction

Infants’ emerging ability to manually interact with objects creates new possibilities for exploration and learning [1–3]. Manual skills develop incrementally: immature infants swat and bat at objects before becoming increasingly coordinated and flexible with their hands in the second year of life [4–20]. Manual actions, such as reaching and holding an object, require the dynamic coordination of the entire musculoskeletal system and are shaped by the demands of the task being performed. During development, this poses a considerable challenge: as their musculoskeletal system grows, the infant must develop and adjust their motor skills to a constantly changing body.

Prior research has examined the development of motor skills in infants [8, 11, 21–26], children [27–29], and adults [30–32]. Many of these studies observed that the beginning of learning a motor skill is characterized by the actor limiting the range of specific joints, thereby eliminating redundant degrees of freedom. Such behavior results in a limited range of movement patterns and a consistent behavioral outcome. Once proficiency is achieved, this restriction in the movement’s degrees of freedom is released. Although this idea has been systematically studied in new skill development in adults and is used as the theoretical framework to interpret findings in motor development, there has been limited direct study across development [33], largely due to methodological limitations.

The human motor system—from the brain to the musculoskeletal structure—is highly complex and nonlinear [15, 33–35]; therefore measurement of the stability and flexibility of movement patterns is difficult. One partial solution has been the study of motor development during discrete trial procedures in which a restrained or supported infant is presented with a motor task such as reaching towards an appearing target. However, motor development occurs in more naturalistic environments and contexts, conferring more possibilities for action than those afforded in tightly controlled experimental tasks. Moreover, recent advances in wearable sensors have allowed us to capture the increasing sophistication of manual behavior in older infants during naturalistic and free-flowing play contexts. Manual play with objects in these contexts is developmentally related to tool use [27, 36, 37], visual object recognition [3, 38, 39], and language [3]. A central contribution of the present study is a new method for estimating spatial-temporal modes of behavior (the shape and size of an attractor region) in the space of all possible hand movements (the state space). We show that, during bouts of manual actions with objects, infants traverse a constrained trajectory in the state space of movement patterns and that the size of their attractor region increases with age: suggesting increased flexibility in manual action patterns.

Our approach was motivated in part by Thelen et al.’s [40] longitudinal study of reaching from onset through the first year of life. Collecting dense recordings of limb movements, Thelen et al. observed the patterns of movement that led up to the emergence of the skill of reaching. Because of the high-dimensional space of the intrinsic dynamics of movements, each successfully produced reach appeared to be unique in its movement patterns. To reduce the dimensionality of kinematic data, Thelen et al. constructed a phase portrait by continuously plotting the relation between movement displacement and velocity. These low-dimensional geometric portraits of patterns of movement revealed stable modes of behavior across reaches and infants. Here, concentrating on the free-flowing actions of reaching for and manually acting with objects during play in older infants, we adapted a novel quantitative protocol for estimating attractor regions [41] across a probabilistic state space akin to a phase portrait.

This quantitative protocol allows us to investigate a number of questions about how manual behaviors change across age and during specific types of actions like reaching and producing manual actions with objects in a free-flowing toy play task. First, little is known about how the motor system changes across age in contexts that are not constrained by discretized trials with specific tasks given by experimenters. Our analysis estimates (1) a probabilistic state space of possible hand movements and (2) an attractor region. The estimated attractor region comprises manual actions that encompass normal modes of spatial-temporal movements that share the same areas in the probabilistic state space. In other words, given all of the possible spatial-temporal movements an infant can make with their hands, movements inside of the attractor region are the most similar movements and movements outside of the attractor region are the least similar movements. The size of the attractor region for any given infant indexes the flexibility of the manual action system, such that a larger region equates to a more flexible system because a larger region comprises more typical hand movements in the state space of all possible hand movements. One of our main hypotheses is that as infants become older, their manual action system becomes more flexible–as indexed by larger attractor regions. We call this hypothesis the *developmental hypothesis*. Our second hypothesis is that manual actions with objects will more often be located in the attractor region of the state space of all possible movements. We call this hypothesis the *attractor hypothesis* because the action of manually acting with an object is an attractor that brings the behavior into the attractor region. Given that our quantitative protocol is novel, testing the attractor hypothesis is important to show that the method is sensitive to changes in manual actions with and without objects.

## 2. Methods

### 2.1. Participants.

A total of 43 parent-infant dyads participated in the current study. Dyads could participate in a maximum of 6 sessions, from age 9 months to 24 months in three-month increments. This is an age range known for rapid development in sensorimotor behaviors [34]. The current dataset encompasses a total of 131 sessions (see Table 1). A total of 3 participants completed all 6 sessions from 9 until 24 months of age and each participant on average completed 3 sessions (*SD*=1.25). Attrition rates were impacted by a number of factors such as the family moving away from the area or missing a session due to being sick.

### 2.2. Stimuli.

There were three sets of three unique novel toys that were used as stimuli. Each toy was a simple shape of uniform color (red, blue, or green) and similar in size (288 cm^3^) and weight (95.25 g). Toys were made from various materials like plastic, hardened clay, aggregated stone, or cloth. Ordering and counterbalancing of stimuli sets occurred for each age group, and, at any one time, one set of three toys was on the tabletop.

### 2.3. Experimental Room.

**Figure 1:**
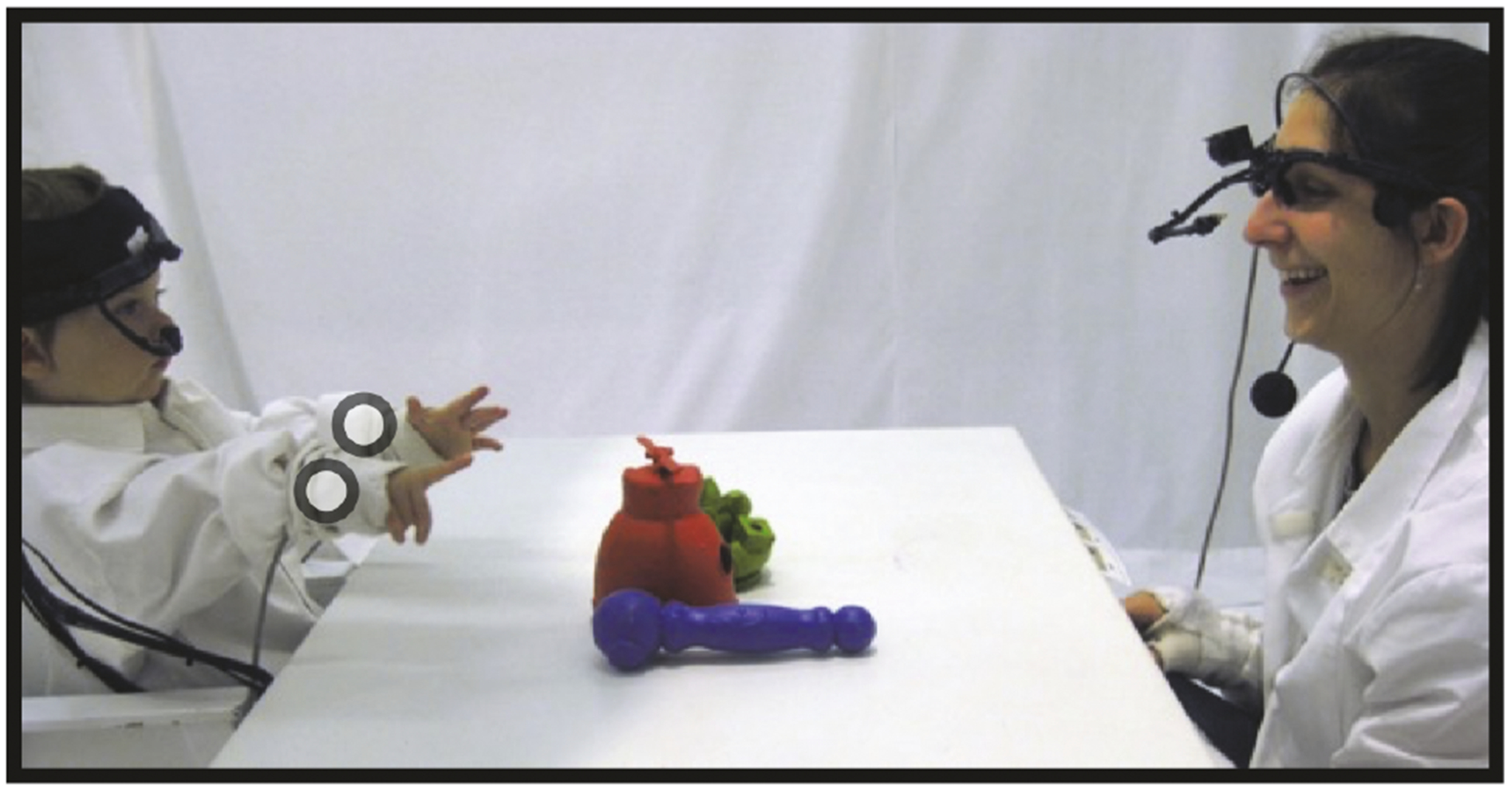
Experimental setup. A parent and their infant sit across a table with toys. Motion sensor placements for the left hand and right hand. Circles represent approximate location and are not to scale.

Infants and parents sat across from each other at a small white table (61 cm × 91 cm × 64 cm). Parents were seated on the ground and infants were seated on a chair that made their eyes, head, and hands approximately the same distance from the table as their parents’ (Figure 1). Infants and parents wore head-mounted eye-trackers and motion sensors affixed to both wrists (Figure 1) and the head. Data collected from the eye-trackers and head-mounted motion sensors were not used in the current study. A Liberty motion tracking system (Polhemus) was used with one sensor embedded in the infant’s headband and two sensors embedded in custom-made gloves that were near the infant’s left and right wrists. The gloves where fabricated to fit around the wrist and act as a wrist cuff, which did not constrain manual actions nor dexterity. Each sensor generated 6 degrees-of-freedom data:3D positional coordinates (x, y, and z) and 3D rotational orientations (roll, pitch, and yaw) of the head and two hands relative to source transmitters centered above the table. The sampling rate for each sensor was 240 Hz but was downsampled to 60 Hz. All analyses described in the current paper were conducted using 3D positional coordinates. High-resolution cameras (30 Hz) were mounted above the table for a bird’s-eye view and also in corners of the room to capture infant and parent perspectives. Video recordings were used in subsequent coding for manual action behavior.

### 2.4. Procedure.

Once the eye-tracking and motion sensors were securely affixed to the infant and parent, an experimenter placed a set of toys on the table and the play session began. Parents were instructed to play naturally with their infant. After approximately 90 seconds of play, an experimenter replaced the toys with a different set and the next trial began. This procedure was repeated and dyads completed up to four trials for a maximum of six minutes of play. Not all dyads completed all of the trials and, therefore, not all total play sessions were six minutes in duration. On average, participants completed 2.77 trials per session (*SD* = 0.56) for an average session duration of 5.60 minutes (*SD* = 1.31) per dyad.

### 2.5. Data Processing and Coding

#### 2.5.1. Manual Action with Object.

Using video recordings from the high-resolution camera, the infants’ manual actions with objects were manually coded and recorded at a sampling rate of 30 Hz by trained research assistants using a custom coding program. Manual actions with objects were defined as manual behavior that included holding and intentional manual actions like touching and fingering. A second coder coded 9 infants’ manual actions from a previous study using the same experimental design with high reliability: Kappa score of 0.96. Proportion of time in manual actions with objects was defined by dividing the total duration of time spent in bouts of manual actions with objects by the total session time. For each session, the preferred hand was identified as the hand with the higher proportion of session time in bouts of manual actions with objects.

#### 2.5.2. Motion Data Processing.

For each of the three sensors, Euclidean distance was computed from the three-dimensional position data to reduce the dimensionality to one dimension.

## 3. Results

### 3.1. The Development of Manual Actions with Objects Behavior in Infants.

The present study examined instances of singlehanded manual actions with objects in infants from 9 until 24 months of age. To determine if there were differences across the preferred hand of the infant, we identified the infant’s preferred hand by calculating the amount of time using manual actions with objects for each hand. The hand with the greater amount of single-handed manual actions with objects was defined as the preferred hand. The properties of manual actions with objects are described in Table 2. We constructed linear mixed effects (LME) models for each effector (two effectors: preferred hand and nonpreferred hand) and for each age. The duration, proportion, and frequency of manual actions with objects were the dependent measures in the LME models. Infant identity was included as a random effect. Fixed effects for LME models included infant age and bout type. Tukey’s Honestly Significant Difference tests were used when multiple comparisons were tested. Duration of bouts of manual actions with objects differed across age (*F*[5, 200]=2.81, *p*=.02), and specifically bouts of manual actions with objects at 9 months (*M*=2.03 seconds, *SD*=3.70) were longer than bouts of manual actions with objects at 24 months (*M*=1.48 seconds, *SD*=2.68), *p*=.03. Duration of manual actions with objects bouts was longer for the preferred hand (*M*=2.00 seconds, *SD*=1.80) compared to the nonpreferred hand (*M*=1.22 seconds, *SD*=0.93, *F*[1, 200]=2.81, *p*=.02). Proportion of time in bouts of manual actions with objects was different across age (*F*[5, 200]=2.43, *p*=.04), but post hoc comparison suggested these differences were nominal. Proportion of time in bouts of manual actions with objects for the preferred hand (*M*=0.17, *SD*=0.07) was higher compared to the nonpreferred hand (*M*=0.06, *SD*=0.04), *F*(5,200)=233.75, *p*<.001. Frequency of bouts of manual actions with objects was different across age (*F*[5, 200]=2.81, *p*=.02), but post hoc comparison suggested these differences were nominal. Frequency of bouts of manual actions with objects for the preferred hand (*M*=5.92 bouts per minute, *SD*=2.46) was higher compared to the nonpreferred hand (*M*=3.73 bouts per minute, *SD*=2.28), *F*(5,200)=62.91, *p*<.001. Overall, the preferred hand had longer and more frequent bouts of manual actions with objects compared to the nonpreferred hand.

### 3.2. The Development of Hand Velocity and Displacement.

**Figure 2:**
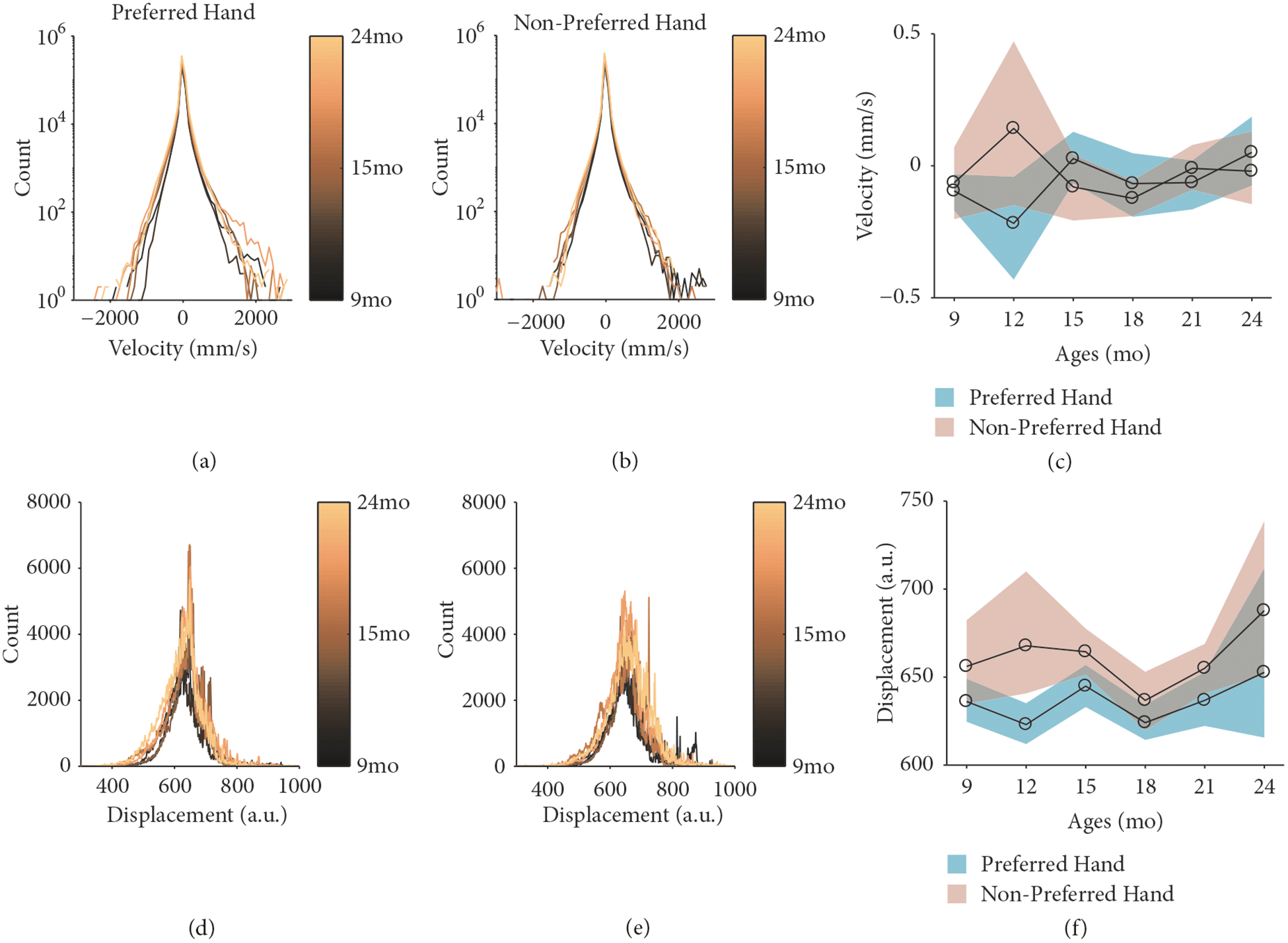
The development of manual actions with objects dynamics. Histograms for the velocity (a, b, c) and displacement (d, e, f) of the preferred (a, d) and nonpreferred (b, e) hand. Colors indicate age group, with lighter shades indicating older children. The average velocity (c) and displacement (f) for the preferred (turquoise) and nonpreferred (beige) hand. Shaded region indicates the 95% confidence interval.

To understand how the dynamics of hand movements developed over time, we first collapsed the x, y, and z coordinates of the preferred and nonpreferred hand’s position by calculating their Euclidean distance, also termed displacement (Figures 2(d) and 2(e)). In other words, displacement is a measure of hand position reduced from the x, y, and z coordinates into one value. From the displacement of each hand sensor, we were able to calculate positional velocity (Figures 2(a) and 2(b)). For the preferred (turquoise) and nonpreferred (beige) hand, the average developmental trajectories of positional velocity and displacement are plotted in Figures 2(c) and 2(f), respectively, along with the 95% bootstrapped confidence interval. At a session level, we observed no significant developmental differences in displacement (*F*[5, 208]=0.61, *p*=.69) or velocity (*F*[5, 208]=2.09, *p*=.07) of the preferred and nonpreferred hand. To determine whether there was a change in the interaction between displacement and velocity–the actual dynamics of hand movements–we characterized each hand as a phase portrait by creating a 2-dimensional state space comprised of the displacement and velocity values from each hand. In the next section, we will go through each step of the quantitative protocol.

### 3.3. Estimation of the Attractor Region from Phase Portraits.

**Figure 3:**
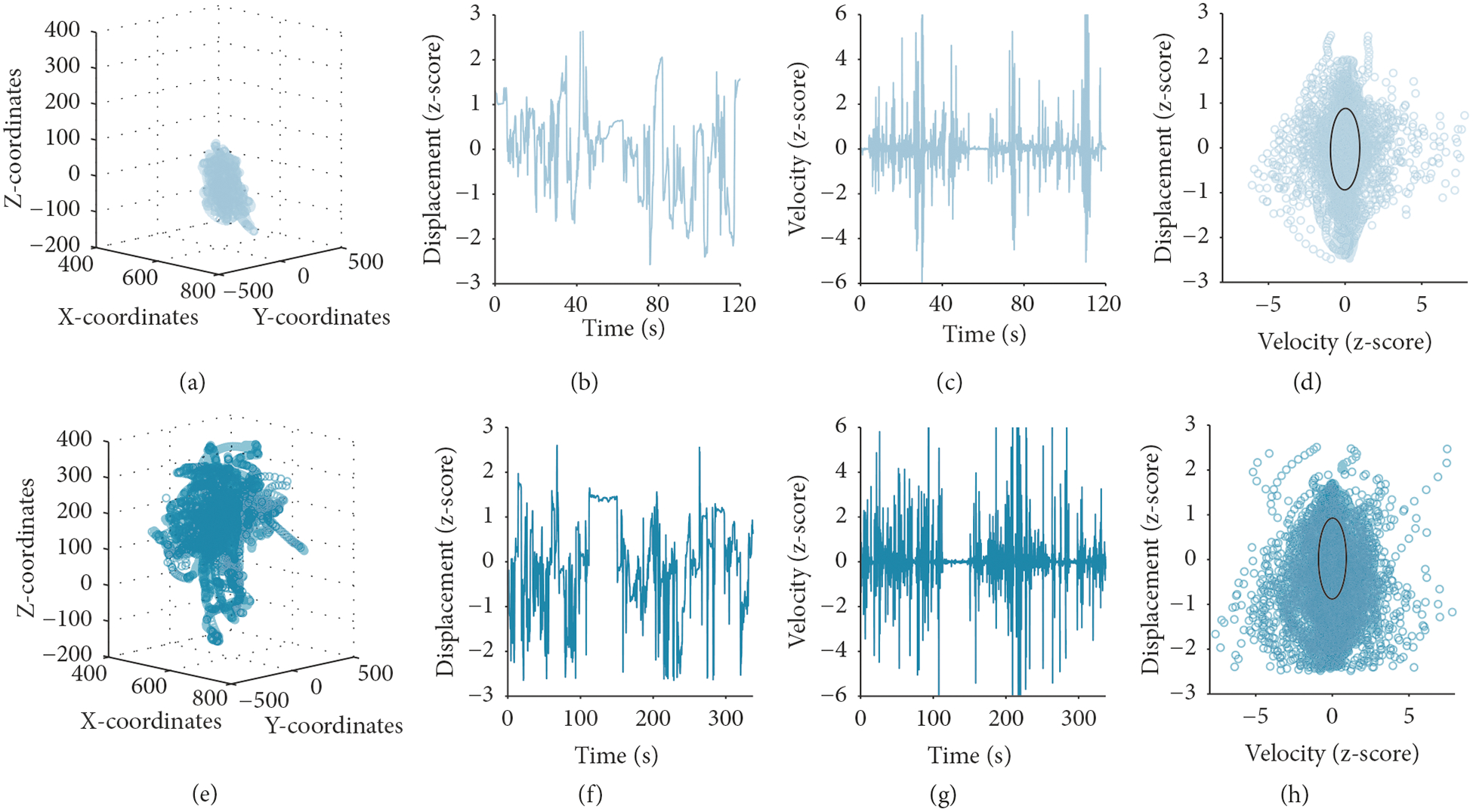
Exemplar of preferred hand movement for the same child at 9 months (a–d) and 24 months (e–h) of age. (a, e) Session data for preferred hand position. (b, f) Euclidian distance for the preferred hand position data. (c, g) Positional velocity derived from the Euclidian distance of preferred hand position. (d, h) Phase portraits of preferred hand velocity (x-axis) against displacement (y-axis). The black ellipse represents the calculated Gaussian fit attractor for the phase portrait.

Prior research has leveraged the dense sampling of cardiac activity, respiration, and body movement to estimate the attractor dynamics of the autonomic nervous system in adult marmoset monkeys while they vocalize [41]. Here we extend the analyses to movement variables from human manual actions in order to capture features of the attractor region for hand movements and any developmental change to these features. We estimated the attractor regions for hand movements by fitting a multivariate Gaussian distribution to the covariance matrix of the hand position data, as follows. The attractor region was estimated on a session-by-session basis for each infant. We first calculated the Euclidian distance between the x, y, and z coordinates for the entire session. Data points that were greater than 2.5 standard deviations away from the mean of the Euclidian distance measure were identified as outliers. We then calculated the velocity of the Euclidian distance and then removed all outlying data points. To control for differences in the location of the infants across sessions and to control for the developmental change in body growth, such as arm length, we z-scored the Euclidian distance and positional velocity measurements. From these normalized Euclidian distance and positional velocity measurements, we can plot the phase portraits for each infant’s preferred and nonpreferred hand for every session (Figure 3). We then calculated the covariance matrix of the z-scored positional velocity and Euclidian distance measurements using cov in Matlab. We fit a multivariate normal Gaussian distribution to the data and calculated the contours encompassing the 50th percentile of the distribution. For each Gaussian fit, we calculated the longest distance along the x-axis (velocity), the longest distance along the y-axis (displacement), and the area of the Gaussian. The area of the Gaussian was considered the size of the attractor region. All possible movements in the x-axis and the y-axis represent the probabilistic low-dimensional state space of movements.

### 3.4. Hypotheses for Attractor Regions of Manual Actions.

The method described above allows for specific questions about how the attractor regions of manual actions change over development and during specific types of behaviors. For example, does the size of the attractor region change throughout the first two years of life? Infants use increasingly more complex manual actions throughout development [25, 36, 42–44]. We expect more flexible manual actions to be produced by hand movements with larger ranges of displacement and velocity. Therefore, our developmental hypothesis is that the attractor regions should increase in area across the developmental period measured, from 9 months until 24 months of age. Increases in the area of the attractor region would suggest a more flexible system of manual actions. A larger attractor region represents a larger range of movement states in the overall state space of movements encompassed by the attractor.

Another feature of our method for estimating attractor regions is that we can investigate the overall amount of time spent inside of the attractor region during specific types of behaviors. For example, what is the proportion of time spent inside of the attractor region during manual actions while manual acting with an object relative to when not manually acting with an object? Our attractor hypothesis is that manually acting with an object is an attractor that moves the manual action system into the attractor region. The behavior of manual actions with objects encompasses many different types of object manipulations. Despite the diversity of object manipulations an infant can perform during manual actions with objects, we expect that the low-dimensional dynamic behavior as observed through the attractor dynamics framework will uncover similar patterns across bouts of manual actions with objects. This is similar to what Thelen et al. [40] observed in reaching: high-dimensional movements were highly variable during reaching, but when observed in low-dimensional phase portraits, the behaviors were actually quite similar showing evidence for a stable limit cycle. Specifically, the attractor hypothesis would suggest that (1) the manual action system spends more time inside of the attractor state during bouts of manual actions with objects and (2) the manual actions with objects are what moves the manual action system into the attractor region.

### 3.5. The Development of Attractor Regions.

**Figure 4:**
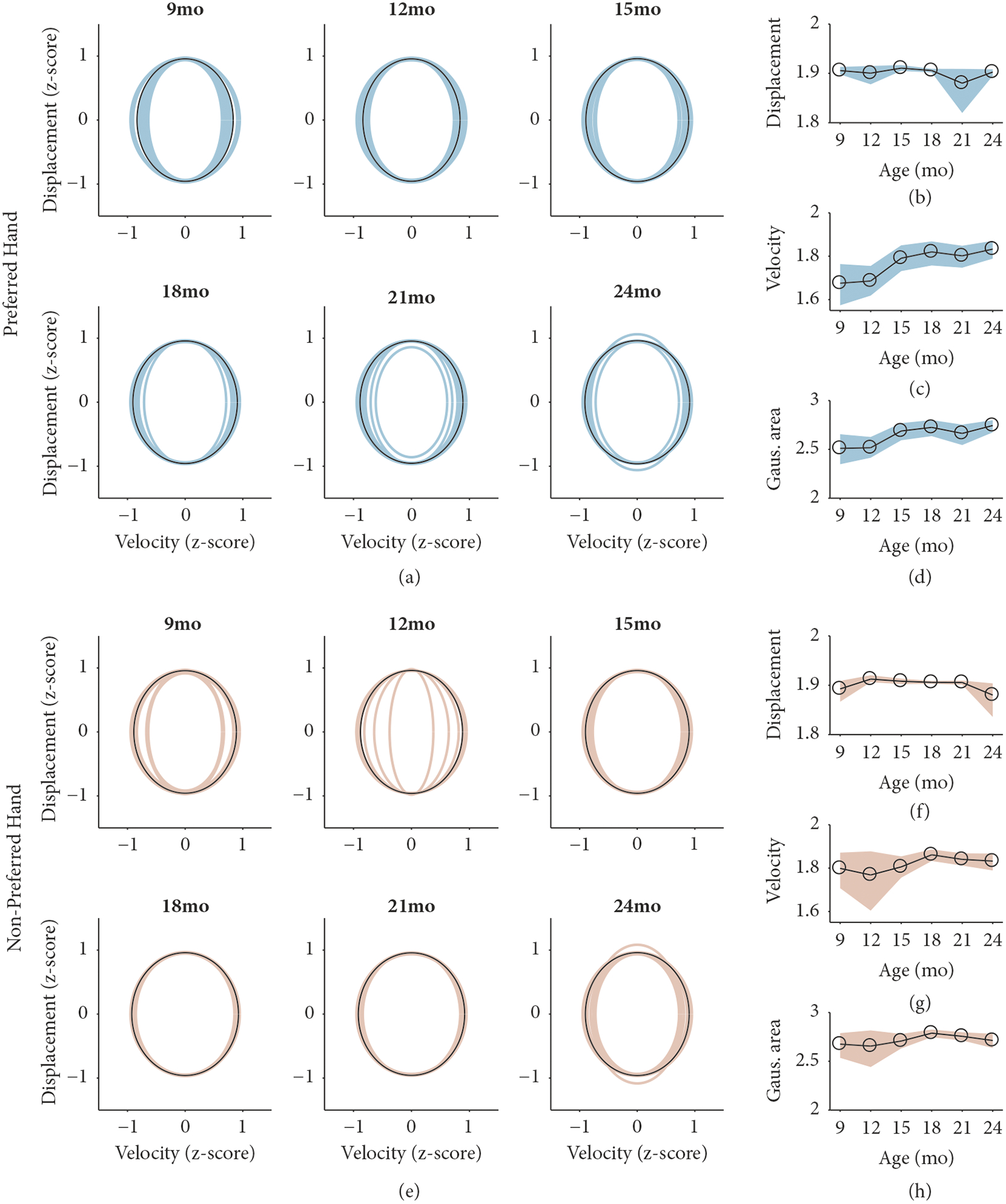
Analyses of the calculated Gaussian fit attractor regions across development. (a, e) The resulting attractor regions for the preferred hand (a, turquoise) and nonpreferred hand (e, beige) based on the phase portrait for each participant in the study. The average attractor region is plotted in black. The x-axis represents z-scored velocity while the y-axis represents z-scored displacement. (b, f) The development of the longest line parallel to the y-axis bounded by the attractor region for the preferred hand (b) and nonpreferred hand (f). (c, g) The development of the longest line parallel to the x-axis bounded by the attractor region for the preferred hand (c) and nonpreferred hand (g). (d, h) The development of the area of the attractor region for the preferred (d) and nonpreferred (h) hand. (b–d, f–h) Black circles indicate the average for each age group while the shaded region indicates the bootstrapped 95% confidence intervals.

The phase portraits in Figures 3(d) and 3(h) demonstrate the breadth of data along two axes: displacement and velocity. The fitted Gaussian attractor regions were unrestrained and had no prior conditions for fitting, besides being centered to the mean of the entire session’s data and bounded by the covariance matrix and 50th percentile of the session. Thus, attractors could be tilted and were not necessarily aligned to the vertical and horizontal axes. The nontilted attractor regions are plotted in Figure 4(a) (preferred hand) and Figure 4(e) (nonpreferred hand). To determine whether the estimated attractor regions captured meaningful developmental change, we sought to measure three features of the attractors over developmental time: the range of (1) velocity and (2) displacement and (3) the area of the attractor. We measured the greatest range of velocity and displacement for each attractor by calculating the longest vertical (displacement, Figures 4(b) and 3(f)) and horizontal (velocity, Figures 4(c) and 4(g)) line that could be drawn within the bounds of the attractor. The area of each Gaussian attractor region was plotted across development for preferred (Figure 4(d)) and nonpreferred (Figure 4(h)) effectors.

We constructed LME models for each effector (two effectors: preferred hand and nonpreferred hand) and for each phase portrait property (three properties: displacement axis, velocity axis, and area), accounting for nine total LME models. Fixed effects for these models included infant age in months. Tukey’s Honestly Significant Difference tests were used when multiple comparisons were tested.

For the preferred hand, there were no age differences for the displacement axis, *F*(5,83)=0.70, *p*=.62. There were age differences for the velocity axis (*F*[5, 83]=4.87, *p*<.001) and for area (*F*[5, 83]=4.16, *p*=.002), suggesting that there were increases in both properties across age. For the velocity axis, the range of the velocity axis was significantly smaller at 9 months (*M*=1.68, *SD*=0.23), compared to 18 months (*M*=1.82, *SD*=0.13, *z*=3.18, *p*=.02), 21 months, (*M*=1.80, *SD*=0.13, *z*=2.98, *p*=.03), and 24 months (*M*=1.83, *SD*=0.11, *z*=3.42, *p*=.008). Attractor region area was significantly smaller at 9 months (*M*=2.51, *SD*=0.36), compared to 18 months (*M*=2.73, *SD*=0.19, *z*=2.93, *p*=.04) and 24 months (*M*=2.74, *SD*=0.16, *z*=3.65, *p*=.004). Total area at 12 months (*M*=2.52, *SD*=0.24) was significantly smaller than total area at 24 months, *z*=3.19, *p*=.02. For the nonpreferred hand, there were no age differences for the displacement axis (*F*[5, 83]=1.54, *p*=.19), the velocity axis (*F*[5, 83]=0.91, *p*=.48), or area, *F*(5,83)=0.88, *p*=.50.

Overall, these results suggest that the manual action system becomes more flexible across the first few years of life, and this depends on hand preference. As indicated by an increase in the size of the attractor region throughout infancy for the preferred hand, the manual action system of the preferred hand becomes more flexible. However, we did not observe such a trend for the nonpreferred hand.

### 3.6. Manual Action with Objects: An Object Is an Attractor.

**Figure 5:**
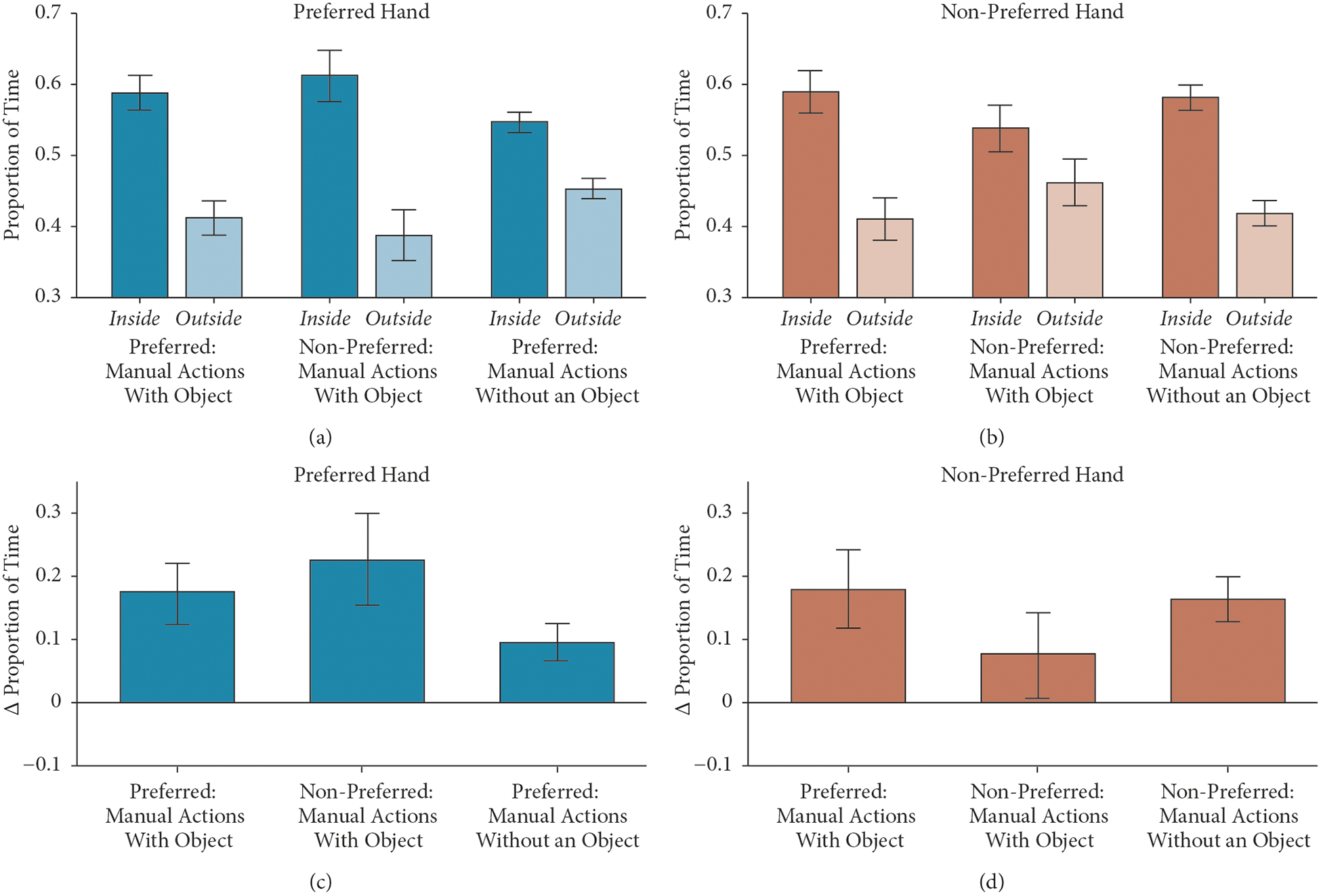
Manual actions with objects inside and outside of the attractor region. (a, b) The proportion of time inside and outside of the attractor region for the preferred (a, turquoise) and nonpreferred (b, beige) hands for three types of events: manually acting with an object with the preferred hand, manually acting with an object with the nonpreferred hand, and no manual action with an object. Lighter shades indicate proportion of time spent outside of the attractor. Error bars indicate the bootstrapped 95% confidence interval. (c, d) The difference in proportion of time inside and outside of the attractor for each event type for preferred (c, orange) and nonpreferred (d, green) hands. Error bars indicate the bootstrapped 95% confidence interval.

To determine the amount of time spent in typical or less typical modes of behavior during manual actions, we computed the relative proportion of time inside or outside of the attractor region for the preferred and nonpreferred hands during bouts when (1) the hand was manually acting with an object, (2) the other hand was doing manual actions with an object (e.g., relative proportion of time the preferred hand is inside and outside of the attractor ellipse when the nonpreferred hand is manually acting with an object), and (3) neither hand is manually acting with an object (Figures 5(a) and 5(b)). If manual actions constrain body movements, we expect higher proportions of each hand inside the attractor region during bouts of manual actions with an object (of either the same or the other hand), relative to bouts when neither hand is manually acting with an object (Figures 5(c) and 5(d)). See Table 1 for bout properties of manual actions with objects for the preferred and nonpreferred hands.

We constructed LME models for each effector (two effectors: preferred hand and nonpreferred hand) and for each type of bout (the same hand manually acting with an object, other hand manually acting with an object, and no manual actions with an object). Because we are interested in the relative proportion of time inside and outside of the attractor region, we computed a delta index, subtracting the total amount of time outside of the region from the total amount of time inside of the region. A positive delta index indicates more time inside of the region relative to outside of the region. The delta index was the dependent measure in the LME models. Fixed effects for LME models included infant age and bout type. In preliminary models, we included infant age as a fixed effect but observed no significant differences, and we therefore omitted infant age in all reported analyses.

For the preferred hand, we constructed two LME models. In the first model, the delta index of the preferred hand was the dependent measure and bout type (preferred hand manually acting with an object, nonpreferred hand manually acting with an object, and no manual action with an object) was the fixed effect. We observed a significant main effect of bout type, *F*(1,260)=6.48, *p*=.002. We observed that the delta index for the preferred hand when the preferred hand was manually acting with an object (*M*=.18, *SD*=.28) was marginally higher compared to bouts of not manually acting with an object (*M*=.09, *SD*=.17), *z*=−2.26, *p*=.06. We also observed that the delta index for the preferred hand when the nonpreferred hand was acting upon an object (*M*=.22, *SD*=.40) was significantly higher compared to bouts of not manually acting with an object, *z*=3.56, *p*=.001.

We constructed a second model to test for overall differences in delta indices for the preferred hand, when either hand was manually acting with an object compared to bouts of not manually acting with an object. In the second model, the delta index was the dependent measure and bout type (either hand manually acting with an object, no manual action with an object) was the fixed effect. We observed a significant main effect of bout type (*F*[1, 261]=11.24, *p*<.001), suggesting that the delta indices for the preferred hand during bouts of either hand manually acting with an object (*M*=.20, *SD*=.35) were higher compared to bouts of not manually acting with an object (*M*=.09, *SD*=.17).

For the nonpreferred hand, we constructed two LME models. In the first model, the delta index of the nonpreferred hand was the dependent measure and bout type (preferred hand manually acting with an object, nonpreferred hand manually acting with an object, and no manual action with an object) was the fixed effect. We observed a significant main effect of bout type, *F*(1,260)=3.67, *p*=.03. We observed that the delta index for the nonpreferred hand when the preferred hand was manually acting with an object (*M*=.18, *SD*=.35) was higher compared to when the nonpreferred hand was manually acting with an object (*M*=.8, *SD*=.39), *z*=−2.51, *p*=.03. We also observed that the delta index for the nonpreferred hand when the nonpreferred hand was manually acting with an object was marginally lower compared to bouts of not manually acting with an object, *z*=−2.13, *p*=.08.

Similar to what was done for the preferred hand, we constructed a second model to test for overall differences in delta indices for the nonpreferred hand, when either hand was manually acting with an object compared to bouts of not manually acting with an object. The main effect of bout type was not significant (*F*[1, 260]=1.01, *p*=.32), suggesting that the delta indices for the nonpreferred hand during bouts of either hand manually acting with an object (*M*=.13, *SD*=.37) were not different from indices during bouts of not manually acting with an object (*M*=.16, *SD*=.20).

These results suggest that when the preferred hand is manually acting with an object, the manual action system–across both hands–is more constrained in the spatial and temporal dimensions. Moreover, when the nonpreferred hand is manually acting with an object, the nonpreferred hand is more likely to be in less probable locations in the state space of possible hand movements. Overall, these results suggest that the preferred and nonpreferred hands have different modes of spatial-temporal behaviors during bouts of manual actions with objects.

### 3.7. The Attractor Dynamics of Manual Actions with an Object.

**Figure 6:**
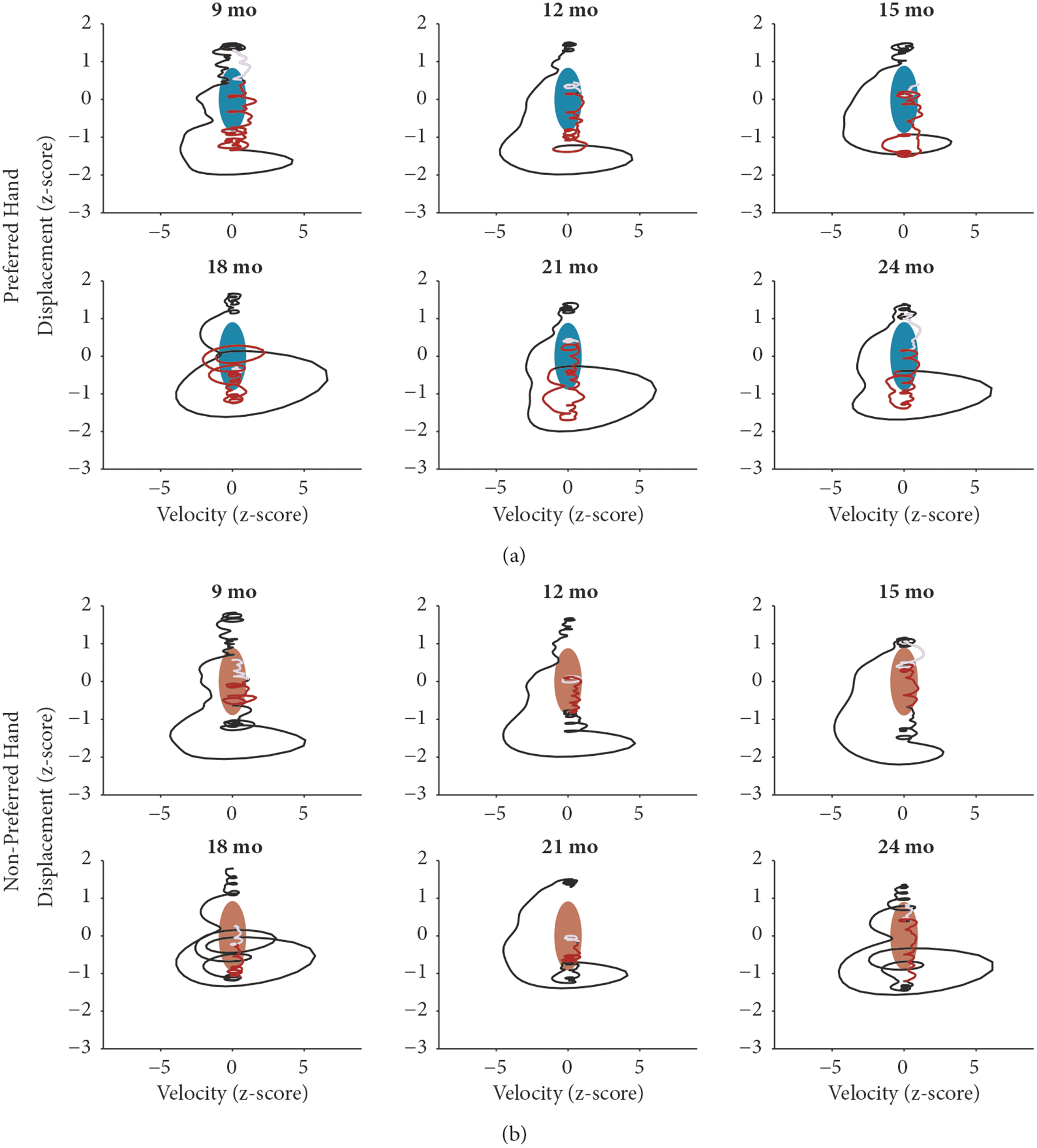
The attractor dynamics of manual action with objects. (a, b) The average attractor region for each age group for the preferred (a, turquoise) and nonpreferred (b, beige) hand. Line indicates the average z-scored velocity and z-scored displacement for 3 seconds before the onset of manual action and 5 seconds after manual action’s onset. The black line indicates the time period 3 seconds before the onset of manual action. The red line indicates the manual action behavior with its duration equal to the average duration of manual action for that age group. The gray line indicates the action’s offset.

We next sought to determine how the average movement trajectory of hand position during manually acting with an object related to our estimated attractor regions. We took the position of the preferred and nonpreferred hand 3 seconds before and 5 seconds after the onset of a manual action with an object. This resulted in a total of 11,360 instances of manual actions with objects across all subjects and age groups with an average of 1,893 instances of manual actions with objects per age group (*SD* = 552). For each instance of manual actions with objects we calculated the Euclidean distance of the x, y, and z coordinates as well as the velocity of the Euclidian distance. We then averaged the Euclidean distance and velocity for each age group and z-scored the resulting average. For each age group, we plotted the z-scored average displacement and velocity measures against the average attractor region for the preferred (Figure 6(a)) and nonpreferred (Figure 6(b)) hands.

Across all ages, the dynamics of manual actions with objects appear remarkably similar. Beginning three seconds before the onset of manual action (Figure 6, black line), there are consistent excursions around the state space before a gradual return into the attractor region once a bout of manual actions with objects begins (Figure 6, red line). For the duration of the bout of manual actions with objects, the trajectory largely stays within the attractor region, even until after the manual action has ended (Figure 6, gray line). This dynamic is consistent across both preferred and nonpreferred hands and across age groups, suggesting the low-dimensional trajectories through the state space before, during, and after manual actions with objects do not differ much during development.

## 4. Discussion

The current study introduced a novel analytical paradigm for estimating attractor regions of manual actions. The paradigm was applied to a large longitudinal corpus of hand movements during infant-caregiver toy play. We observed that the size of attractor regions increased throughout development, suggesting that the manual action system becomes more flexible throughout development. We also observed that, in a state space of possible movements, hand movements from the preferred hand during bouts of manual actions with objects were more likely to be in the attractor region.

The proposed *developmental hypothesis* suggests that attractor regions should increase in area throughout the first two years of life. We observed partial evidence for this hypothesis. Across development, we demonstrated that the attractor region for the preferred hand increases in both area and range of velocity (Figures 4(c) and 4(d)). The nonpreferred hand, in contrast, showed no developmental change along velocity, displacement, or area (Figures 4(f)–4(h)). The observed increases in the area of the attractor region for the preferred hand suggest a more flexible system supporting its actions. A larger attractor region covers a larger area of displacement and velocity, facilitating a more diverse range of movements. Throughout the first few years of life, infants perform increasingly complex toy play behaviors [25, 36, 42–44]. Our results suggest that these complex behaviors are supported by a manual action system that is becoming more flexible. It is important to note the distinction between a flexible system and a more controlled system. Our results point specifically to the *flexibility* of a system, whereas other methods have been successfully implemented to measure control, which, in the same topology as our phase portraits, would be in the form of observing stable limit cycles [40, 45].

The proposed *attractor hypothesis* suggests that manual action with an object is an attractor and therefore we should (1) observe the manual action system to spend more time inside the attractor region and (2) observe that the manual action with an object is what moves the manual action system into the attractor region. We observed that when either hand was manually acting with an object, the preferred hand movements were more likely to be inside the attractor region than outside of the attractor region. This observation provides partial support for the attractor hypothesis. We also observed that the nonpreferred hand movements were more likely to be inside of the attractor region when the preferred hand was manually acting with an object compared to when the nonpreferred hand was manually acting with an object. Previous research has shown that as the motor system develops, the so-called motor overflow – one limb showing similar behavior as the other limb during specific actions – decreases, which has been suggested to mark the emergence of more specialized motor actions such as unimodal manual actions [46, 47]. Our results do not shed any new light on the evidence for motor overflow but rather point to the increased complex behavior such as unimodal manual actions and role-differentiated bimodal action that become more prevalent going into the second year of life [23] (Gold-field and Michel, 1986; Kimmerle, Mick, and Michel, 1995; Kotwica, Ferre, and Michel, 2008), which are the suggested consequences of the cascading effects of motor overflow. Our current analyses were agnostic as to the exact trajectories of manual actions with objects and did not directly compare the trajectories of each hand. Instead, the increased proportion of time the preferred hand remained in the attractor region during nonpreferred manual actions with objects suggests that the nonpreferred hand’s manual actions with objects still recruit effort from the preferred hand, perhaps implicating a mechanism similar to motor overflow. Further investigation would be necessary to link the observed phenomena with the concept of motor overflow, especially at younger ages when motor overflow has been known to occur.

Finally, when we plot the average trajectory of hand movements during manual actions with objects through the probabilistic state space of movements, we find that manual actions with objects have consistent trajectories that end inside of the attractor region across all age groups. Beginning three seconds before the onset of manual actions with objects, there is an excursion away from the attractor region. The onset of manual actions with objects occurs just before the movement in the state space approaches the attractor region for the preferred hand. For the nonpreferred hand, movements are already in the attractor region at the onset of a manual action. For both preferred and nonpreferred hands, manual actions with objects are characterized by a period of low velocity and little movement along the displacement axis. While this study only looked at instances of single-handed manual actions with objects, it is likely that two-handed manual actions with objects would share similar dynamics.

The functional result of low hand velocity and movement during manual action is the stabilization of the object. Putatively, this would maximize visual information that could be processed from the object while it is in view. While this study did not measure the amount of looking time of the held object, prior research suggests that attention to objects requires sensorimotor coordination that stabilizes body movements and likely facilitates learning [48, 49]. In our framework, it is intriguing to consider attractors from other modalities. For example, does gaze behavior–which occurs at a faster timescale relative to manual actions–push manual actions inside and outside of attractor regions? Alternatively, it is possible that the slower-changing dynamics of manual actions constrain the faster-changing dynamics of gaze behaviors [50]: manual actions with objects are attractors pushing gaze behavior into modes of sustained attention.

This study contributes to a number of areas in the literature. Many previous studies have studied how the motor system reorganizes when learning new skills and how the motor system changes throughout development [8, 11, 21, 22, 25, 26, 28, 36, 42, 43]. However, our study is the first–to our knowledge – to index the development of flexibility of manual action in a natural free-flowing context throughout the first two years of life. By showing that the preferred hand becomes more flexible across development–as observed by increased attractor region size – we add more insight into the developmental trajectory of the manual action system. It should be noted that a limitation of the current paper is that the level of analysis of manual actions with objects is only informative to whether or not manual actions include or do not include an object. Future work needs to determine whether specific types of manual actions with objects, such as holding, touching, and fingering, generate different types of phase portraits across development. Our study also contributes a new method for reducing the dimensionality of behavior down to a phase portrait and then quantifying properties such as the size of an attractor region or the time inside or outside of an attractor region. At the outset of this paper, we discussed Thelen et al.’s [40] conceptual treatment of a phase portrait of reaching behaviors as a motivation for our new method. Although previous research has used phase portraits of specific behavior as a topological space for understanding stable motor behavior [15, 40, 45, 51–55], most of this work focused on periodic behavior (e.g., reiterant speech) and not on quantifying properties of phase portraits constructed from aperiodic behavior like natural free-flowing dyadic toy play. Therefore, the current study provides a novel method for indexing properties of phase portraits assembled from natural behaviors that would not be classified as periodic.

The present study leverages a dense corpus of hand movements during parent-infant play and demonstrates one tractable way to quantitatively define the attractor region for hand movements. We demonstrate developmental changes in the attractor dynamics of the preferred hand, consistent with the emergence of flexible motor behavior. We also demonstrate that the manual action with objects itself occurs within the attractor region of the limb’s movement, a region characterized by low velocity and low speed. This study serves as a first step in quantitatively defining the development and function of attractor dynamics in manual action.

## Figures and Tables

**Table 1:** Mean age and number of participants for each age group.

Age Group	Mean Age in Months (SD)	# Participants/Sessions
9 months	9.63 (0.24)	22
12 months	12.72 (1.08)	18
15 months	15.49 (0.25)	20
18 months	18.65 (0.30)	20
21 months	21.64 (0.23)	25
24 months	24.54 (0.49)	26

**Table 2:** Mean estimates for manual actions with objects properties for the preferred and non-preferred hands (+/−95% CIs in parentheses).

	9 months	12 months	15 months	18 months	21 months	24 months
*Manual actions*						
*with objects duration (s)*						
Preferred	2.03	1.50	1.96	1.71	1.68	1.48
	(1.70,2.39)	(1.31, 1.70)	(1.70, 2.22)	(1.48, 1.95)	(1.45, 1.88)	(1.31, 1.66)
Non-Preferred	1.11	1.04	1.18	1.05	1.02	0.86
	(0.90,1.34)	(0.87,1.23)	(0.97,1.42)	(0.90,1.22)	(0.86,1.17)	(0.74, 0.99)
*Proportion*						
Preferred	0.16	0.17	0.20	0.18	0.14	0.15
	(0.12,0.19)	(0.13, 0.20)	(0.17, 0.24)	(0.16, 0.21)	(0.11, 0.17)	(0.13, 0.18)
Non-Preferred	0.05	0.08	0.07	0.06	0.06	0.05
	(0.04, 0.07)	(0.06, 0.01)	(0.05, 0.01)	(0.05, 0.07)	(0.05, 0.07)	(0.05, 0.07)
*Frequency (per minute)*						
Preferred	5.07	6.60	6.17	6.64	5.02	6.24
	(3.73, 6.51)	(5.32, 7.99)	(5.35, 7.11)	(5.55, 7.82)	(4.26, 5.97)	(5.52, 6.99)
Non-Preferred	3.02	4.69	3.48	3.49	3.51	3.89
	(2.03, 4.16)	(3.46, 5.94)	(2.68, 4.26)	(2.77, 4.33)	(2.73, 4.38)	(3.05, 4.89)
